# Indications, complications and outcomes of minimally-invasive lateral lumbar interbody fusion with anterior column realignment vs. standard LLIF using expandable interbody spacers

**DOI:** 10.3389/fsurg.2024.1455445

**Published:** 2024-12-09

**Authors:** Gregor Fischer, Linda Bättig, Thomas Schöfl, Ethan Schonfeld, Anand Veeravagu, Benjamin Martens, Martin N. Stienen

**Affiliations:** ^1^ Spine Center of Eastern Switzerland, Cantonal Hospital of St. Gallen & Medical School of St. Gallen, St. Gallen, Switzerland; ^2^ Department of Neurosurgery, Cantonal Hospital of St. Gallen & Medical School of St. Gallen, St. Gallen, Switzerland; ^3^ Department of Orthopedic Surgery, Cantonal Hospital of St. Gallen & Medical School of St. Gallen, St. Gallen, Switzerland; ^4^ Department of Neurosurgery, Stanford University, Stanford, CA, United States

**Keywords:** ACR, LLIF, expandable, cage, interbody, outcomes, ELSA, deformity

## Abstract

**Background:**

Anterior column realignment (ACR), using a lateral lumbar or thoracic interbody fusion (LLIF) approach to release the anterior longitudinal ligament (ALL), is a powerful technique to increase segmental lordosis. We here report our experience with the use of expandible LLIF cages for ACR.

**Methods:**

Retrospective, single-center observational cohort study including consecutive patients treated by LLIF using an expandible interbody implant. Patients with ACR were compared to patients without ACR. Our outcomes include adverse events (AEs), radiological (segmental sagittal cobb angle, spinopelvic parameters) and clinical outcomes until 12 months postoperative.

**Results:**

We identified 503 patients, in which we performed LLIF at 732 levels. In 63 patients (12.5%) and 70 levels (9.6%) an expandable cage was used. Of those, in 30 patients (47.6%) and 30 levels, the ALL was released (42.8%). Age (mean 61.4 years), sex (57.1% female), comorbidities and further demographic features were similar, but patients in the ACR group had a higher anesthesiologic risk, were more frequently operated for degenerative deformity and had a more severely dysbalanced spine (all *p* < 0.05). ACR was most frequently done at L3/4 (36.7%) and L4/5 (23.3%), entailing multilevel fusions in 50% (3–7 levels) and long constructs in 26.7% (>7 levels). Intraoperative AEs occurred in 3.3% (ACR) and 3.0% (no ACR; *p* = 0.945). In ACR cases, mean segmental lordosis changed from −2.8° (preoperative) to 16.4° (discharge; *p* < 0.001), 15.0° (3 months; *p* < 0.001) and 16.9° (12 months; *p* < 0.001), whereas this change was less in non-ACR cases [4.3° vs. 10.5° (discharge; *p* < 0.05), 10.9 (3 months; *p* < 0.05) and 10.4 (12 months; *p* > 0.05)]. Total lumbar lordosis increased from 27.8° to 45.2° (discharge; *p* < 0.001), 45.8° (3 months; *p* < 0.001) and 41.9° (12 months; *p* < 0.001) in ACR cases and from 37.4° to 46.7° (discharge; *p* < 0.01), 44.6° (3 months; n.s.) and 44.9° (12 months; n.s.) in non-ACR cases. Rates of AEs and clinical outcomes at 3 and 12 months were similar (all *p* > 0.05) and no pseudarthrosis at the LLIF level was noted.

**Conclusions:**

ACR using an expandible LLIF interbody implant was safe, promoted solid fusion and restored significantly more segmental lordosis compared to LLIF without ALL release, which was maintained during follow-up.

## 1 Introduction

The surgical management of degenerative disc disease (DDD) and adult spinal deformity (ASD) is gradually recognized as a significant healthcare concern, with aging Western societies and rising expectations regarding quality of life (1). Age-related spinal pathologies often present as degenerative processes involving the intervertebral discs, endplates, ligaments, and facet joints (2). Degeneration in combination with loss of muscles and bone density leads to diminished structural support and progressive spinal deformity (2, 3). ASD may result from untreated adolescent idiopathic scoliosis, adult-onset degenerative scoliosis, or primary sagittal imbalance and typically progresses with ageing (1, 4). With exhausted conservative treatment, successful surgical correction of ASD has been shown to significantly enhance affected patients’ quality of life. Several studies have highlighted the direct correlation between the degree of sagittal imbalance and the severity of pain, functional impairment, and reduction in quality of life (5–7) which stresses the importance of restoring a physiological sagittal alignment.

Traditionally, surgical approaches to realign the lumbar or thoracolumbar spine in both coronal and sagittal planes for ASD have relied on posterior open approaches with various osteotomy techniques. Commonly utilized osteotomies include Smith-Petersen osteotomies (SPO) or Ponte osteotomies of the posterior column, or three-column (wedge) resections like pedicle subtraction osteotomies (PSO) or vertebral column resections (VCRs) (7). Although PSOs and VCRs are highly effective in restoring lordosis and rebalance the spine, they are associated with relevant rates of intra- and postoperative complications including excessive blood loss, neurological deficit, instrumentation failure with pseudoarthrosis and adjacent segment disease (1, 8, 9). Over recent years, lateral lumbar interbody fusion (LLIF) has evolved as a minimally-invasive surgical (MIS) technique, which is increasingly utilized for lumbar DDD and ASD (10–12). Historically, MIS techniques have been effective in restoring coronal balance but have met limitations in achieving sagittal correction (13), as the strong anterior longitudinal ligament (ALL) prevents excessive extension of the anterior column. Anterior column realignment (ACR) has emerged to specifically address sagittal imbalance, offering substantial correction of segmental lordosis (SL) comparable to that achievable with PSO. ACR involves release of the ALL through anterior annulotomy and anterolateral discectomy, achieved via a lateral trans- or antepsoas surgical corridor (14, 15). By leveraging the advantages of ALL release and the placement of hyperlordotic lateral cages, ACR enables significant sagittal correction at the targeted level, aiming to restore spinal alignment through a MIS approach.

There is a paucity of studies reporting on results of ACR with the use of expandable LLIF spacers, which we have applied for this indication in the past years. The aim of this study was to review our series of ACR via a LLIF approach and use of an expandable LLIF spacer and analyze intra- and postoperative complications, SL, and the clinical outcomes.

## 2 Methods

### 2.1 Hospital setting

The Cantonal Hospital of St. Gallen, Switzerland is an academic, tertiary teaching-hospital, associated with the Medical School of St. Gallen. It serves a population of approximately 1,000,000 inhabitants. The Spine Center of Eastern Switzerland is formed mutually by 12 board-certified neurosurgeons or orthopedic spine surgeons and seven residents/physician assistants. Use and types of implants are unified. About 1,100–1,300 spine surgical procedures under general anesthesia are performed annually. LLIF with static cages was introduced at our center in November 2011 and is performed about 50–60 times per year on average. The ELSA® Expandable Integrated LLIF Spacer (Globus Medical Inc, PA, USA) as expandable option for LLIF was first used in 09/2018. LLIF procedures were performed by nine senior spine surgeons, or under supervision by fellows or senior residents.

### 2.2 Patient selection & identification

Patient with mobile spinal segments and severe “flat-back deformity”, requiring increase in SL between Th8-L5 were selected for ACR via a LLIF trajectory. The difference between desired and actual SL was calculated for each patient (www.spinebit.io), and deformity correction was simulated with available software for surgical planning (www.surgimap.com). For the segment to be approached, the psoas anatomy and the relationship of the large blood vessels with the approach-side was analyzed to determine suitability of LLIF. In patients requiring substantially more lordosis at the L5/S1 level, either anterior lumbar interbody fusion (ALIF) with a hyperlordotic cage or a transforaminal anterior release (TFAR) (16) was considered in addition or as alternative to LLIF. Similarly, patients with spinal segments fused in kyphosis were considered for PSO or VCR in addition or as alternative to LLIF.

Included procedures encompassed ventro-dorsal, dorso-ventral, and dorso-ventro-dorsal fusion procedures. We identified the unique patient numbers (UPNs) of patients who underwent placement of an ELSA® spacer until January 2024 by reviewing electronic records from our hospital's purchasing department. Additionally, we ensured inclusion of patients who underwent LLIF procedures with an expandable implant by cross-checking the operating program.

### 2.3 Data collection and variables

Three spine surgeons reviewed all patient data and performed measurements of the specified radiological parameters utilizing the Xero Viewer (Version 1.0.0.R812, AGFA HealthCare).

Preoperative patient characteristics, encompassing variables such as the Charlson comorbidity index (CCI) (17), Canadian Clinical Frailty Index (18), and smoking status, were meticulously documented as part of the study protocol. Intraoperative complications such as vascular or nerve injuries, duration of surgery in minutes, and estimated blood loss in milliliters were also recorded during the surgical procedures. Intraoperative complications such as accidental ALL release, vascular or nerve injuries, duration of surgery in minutes, and estimated blood loss in milliliters were also recorded during the surgical procedures.

Postoperative complications at various intervals (at discharge, 3 months, and 12 months post-surgery) were documented using the Therapy-Disability-Neurology (TDN) grading scale (19), providing a comprehensive and patient-centered classification system, offering insights into the severity of adverse events (AEs) across multiple dimensions.

**Figure 1:**
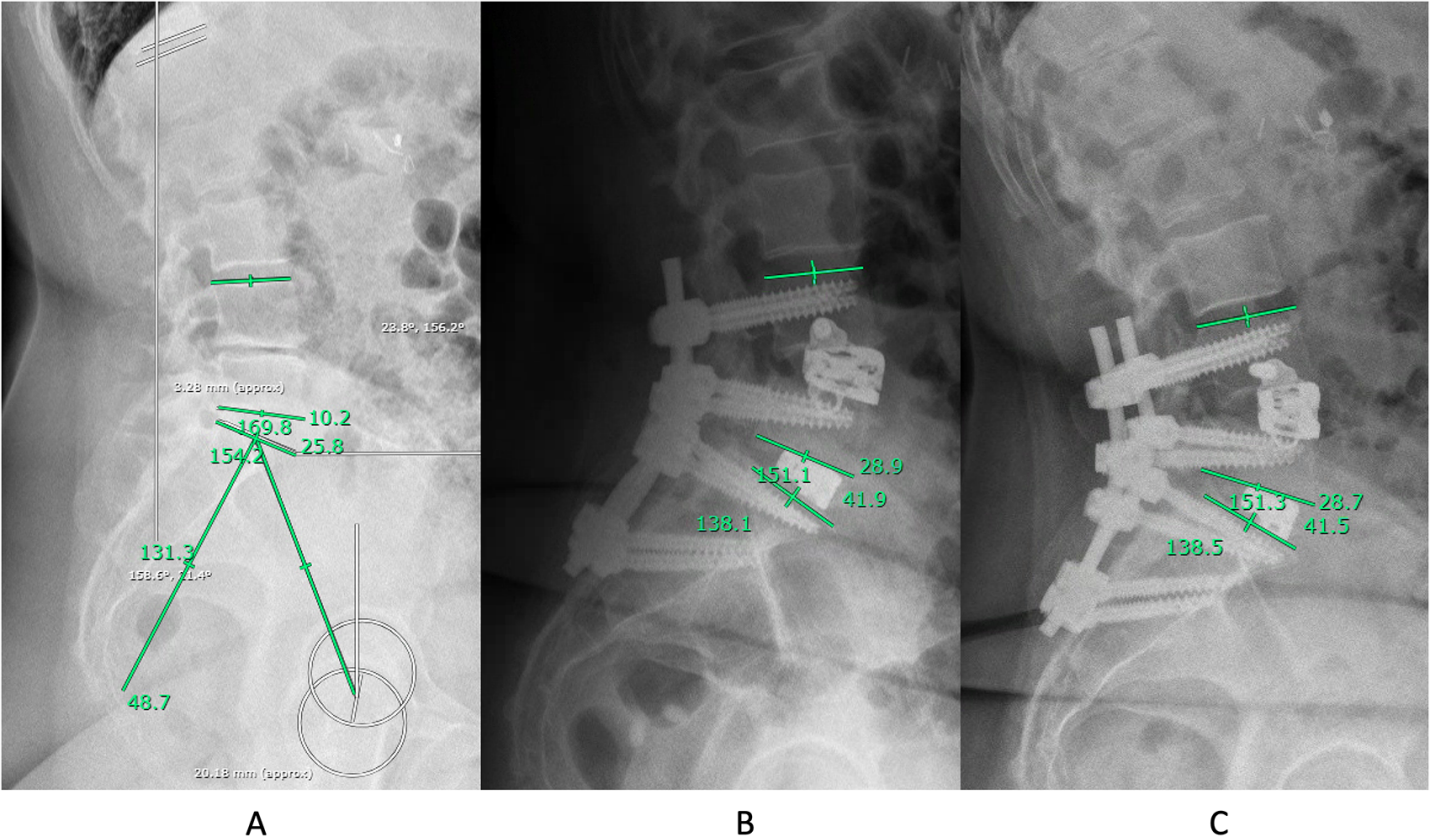
A 60-year-old female with a pelvic incidence of 48.7° presented with advanced degenerative disc disease affecting the L4/5 and L5/S1 segments, including left-sided disc protrusion, and bilateral neuroforaminal stenosis at the L5/S1 level. **(A)** Note the diminished preoperative segmental lordosis (SL) measuring 10.2° at the L4/5 level and 25.8° between L4-S1. According to www.spinebit.io the ideal lordosis between L4-S1 is between 38 and 43°, hence we opted for LLIF at L4/5 with ACR [expandable ELSA®- Cage (5-20°)] and TLIF at L5/S1 with posterior instrumented fusion from L4-S2 (sparing the ilio-sacral joints on purpose). **(B)** Postoperative standing x-rays before discharge, achieving a SL of 28.9° at L4/5 and lordosis of 41.9° between L4-S1. **(C)** Two-year follow-up lumbar x-ray studies indicating stable segmental correction of 28.7° at the L4/5 level and lordosis of 41.5° between L4-S1 with the patient reporting an excellent clinical outcome.

Radiological parameters were measured pre- and postoperative before discharge, as well as at 3- and 12-months on standing/sitting conventional x-ray, conventional whole spine/scoliosis x-ray or EOS® biplane x-ray, as available. Various clinically relevant sagittal parameters were measured, including pelvic incidence (PI), pelvic tilt (PT), lumbar lordosis (LL), thoracic kyphosis (TK), C7 sagittal vertical axis (SVA) and SL. SL was calculated as the angle between the superior endplate of the upper instrumented vertebra and the inferior endplate of the lower vertebra within the segment treated with ACR (Figure 1). We report lordosis as positive, and kyphosis as negative values. The Roussouly type (20) of spinal geometry was determined based on the PI, as the SS was severely altered in this ASD population to compensate for lack of lordosis.

Pseudarthrosis was defined as delayed onset of symptoms with axial or radicular pain weeks to months after attempted fusion surgery with absence of solid posterolateral and intersomatic fusion within the instrumented spinal segment as seen on postoperative imaging, applying the classifications by Lenke et al. (21), Brantigan, and Steffee (22). Diagnosis of pseudarthrosis was based on both the clinical presentation in conjunction with available imaging studies [x-ray, computed tomography (CT) and Single Photon Emission Computed Tomography (SPECT)], after ruling out other causes of persistent pain (23, 24).

### 2.4 Surgical technique

Prior to surgery, preoperative standing x-rays or EOS® biplane x-ray, as well as magnetic resonance imaging (MRI) and/or CT scans, were obtained. These were utilized to assess and determine the most favorable approach side, ensuring the safest trajectory and optimal working plane for the surgical procedure with focus on the location of the anterior vascular structures, the psoas and lumbar plexus anatomy. Posterior instrumented fusion using pedicle screws and rods were undertaken in all cases, either prior to or following the LLIF procedure, usually applying posterior column osteotomies (PCOs) in ACR cases. No standalone LLIF procedures were performed.

All LLIF procedures were performed in lateral decubitus position, employing standard positioning and fluoroscopic targeting techniques. The MaXcess® [NuVasive, Inc., La Jolla, CA (USA)] retractor was used for all procedures in MIS-fashion to keep the incisional length reasonably small (usually 4–6 cm) and mitigate approach-related morbidity. Sequential dilators were passed through the retroperitoneal space with digital guidance & bluntly through the psoas muscle while rotating under evoked directional electromyography (EMG) to map neural structures for levels L2/3 or lower. Considering that the lumbar plexus should lie posterior to the access corridor, retractors were docked as posteriorly as deemed safe and opened until the anterior aspect of the disc space with ALL becomes visible. The anterior vessels were dissected in the adventitial plane with blunt instruments and an ALL retractor was placed, serving as barrier between these structures and the operative workspace while avoiding any folding of veins. After thorough discectomy with contralateral annulus release, trial spacers were inserted and the definitive spacer was chosen with desired dimensions and angulation. Implants ranged from parallel (0°) to anatomical (6° lordosis), lordotic (5–20° lordosis), until hyperlordotic (15–30°) angulation. The definitive spacer was introduced under serial fluoroscopy, opened until initial contact with the adjacent vertebral endplates was made, and was then fixated in one or both vertebrae with appropriately sized screws to reduce the risk of implant migration, particularly given the increased segmental mobility following ALL release. While fixation with a single screw allows greater flexibility for subsequent posterior correction, two screws offer protection against vertebral translation if a full segmental release is performed. The slightly tensioned and isolated ALL was cut at least at 2/3rd of length using a long-handled scalpel or chisel in case of significant anterior osteophytes. Spacers were then expanded further to complete the ALL release by controlled rupture of the remaining fibers and until the desired disc height and SL was achieved.

### 2.5 Statistical analysis

From the total cohort of patients undergoing LLIF with use of an expandable interbody spacer, patients with ALL release were identified (*n* = 30) and compared against patients without ALL release (*n* = 33). Throughout the subsequent sections, the groups are referred to as w ALL release (ACR group) and w/o ALL release (non-ACR group). Baseline demographic variables and surgical characteristics were reported as mean (standard deviation) or count (percent) and compared between the study and control group using *t*-tests and Chi-square tests, as appropriate. To analyze sagittal spinal parameters over time, we compared outcomes in both the study and control groups to their respective preoperative values using paired *t*-tests. We calculated the difference between desired (ideal) LL and observed (actual) LL, as well as PI-LL mismatch at each time point of follow-up, determining the ideal values using a web-based app (http://www.spinebit.io), which is based on the formulas by Le Huec and the European Spine Study Group (25–27). At time of discharge, 3 and 12 months postoperative, complications and clinical outcome according to MacNab was reported and compared between groups using Chi-square tests. Logistic regression models were built for three key variables, (1) any complication until 12 months, (2) any surgical complication until 12 months, and (3) favorable (excellent or good) outcome at 12 months, estimating the odds ratio (OR) and 95% confidence interval (CI) with respect to ALL release. First, univariable models were built to analyze direct relationships. Then, multivariable models were built to adjust for potential confounders. Stata [StataCorp LLC, College Station, TX (USA)] SE v18 for Mac was used. Probability values <0.05 were considered statistically significant.

### 2.6 Ethical considerations

The study (BASEC ID 2023-01343) received approval from the institutional review board (IRB) of Eastern Switzerland. An institutional waiver for informed consent permitted the retrospective collection, analysis, and publication of anonymized patient data.

## 3 Results

### 3.1 Study cohort

We identified a total of 503 patients, among whom we conducted LLIF procedures on 732 levels. Within this cohort, an expandable spacer (Globus Medical, Expandable Integrated Lateral Interbody Spacer: ELSA®) was utilized in 63 patients (12.5%) and across 70 levels (9.6%). Baseline demographics revealed a mean age of 61.4 years and a slight female predominance of 57.1%. The ALL was intentionally released in 30 patients (47.6%) and 30 levels (42.8%). We did not identify any case with accidental, unintended ALL release, hence no patient was excluded for this reason. Statistical analysis revealed no significant differences between the ACR and the non-ACR groups in terms of age, sex, Charlson comorbidity index (CCI), Canadian clinical frailty index, smoking status or Roussouly classification of spinal geometry. Notably, patients in the ACR group exhibited a higher anesthesiologic risk according to ASA risk scale, underwent surgery more frequently for DDD, fusion revision or ASD, and presented with a more severely dysbalanced spine, depicted by lower preoperative LL and higher PT (*p* < 0.05; Table 1).

**Table 1:** Baseline demographic information of patients treated by lateral lumbar interbody fusion using an expandable interbody cage with (w/) or without (w/o) release of the anterior longitudinal ligament (ALL).

Variable	w/ ALL release	w/o ALL release	*p*-value
Age in years	63.8 (12.2)	59.3 (18.3)	0.263
Sex			0.344
Female	19 (63.3%)	17 (51.5%)	
Male	11 (36.7%)	16 (48.5%)	
ASA risk scale			0.008
I	2 (6.7%)	12 (36.4%)	
II	15 (50.0%)	6 (18.2%)	
III	13 (43.3%)	14 (42.4%)	
IV	-(0%)	1 (3.0%)	
Charlson comorbidity index			0.909
0–1	12 (40.0%)	15 (45.5%)	
2–3	8 (26.7%)	8 (24.2%)	
4 or higher	10 (33.3%)	10 (30.3%)	
Canadian clinical frailty index			0.053
Very fit or well	5 (16.7%)	14 (42.4%)	
Managing well or vulnerable	17 (56.7%)	12 (36.4%)	
Mildly or moderately frail	8 (26.7%)	5 (15.1%)	
Severely or very severely frail	-(0%)	2 (6.1%)	
Smoking status			0.343
Active smoker	10 (33.3%)	6 (18.2%)	
Former smoker	2 (6.7%)	4 (12.1%)	
Nonsmoker	18 (60.0%)	23 (69.7%)	
Indication for surgery			0.008
Trauma^a^	4 (13.3%)	17 (51.5%)	
Deformity	9 (30.0%)	7 (21.2%)	
Revision surgery	3 (10.0%)	1 (3.0%)	
Degenerative	12 (40.0%)	4 (12.1%)	
Other	2 (6.7%)	4 (12.1%)	
Spino-pelvic parameters			
Pelvic incidence in°	56.7 (13.9)	49.6 (10.3)	0.024
Lumbar lordosis in°	27.8 (20.1)	37.4 (15.8)	0.038
Pelvic tilt in°	27.3 (8.6)	19.7 (10.6)	0.004
Segmental lordosis in°	−2.8 (13.6)	4.3 (17.1)	0.064
C7 sagittal vertical axis in cm	7.9 (6.6)	8.1 (6.2)	0.935
Roussouly type of spinal geometry			0.552
Type 1 (SS <35°)	3 (10.0%)	4 (12.1%)	
Type 2 (SS <35°)	5 (16.7%)	10 (30.3%)	
Type 3 (35° <SS <45°)	10 (33.3%)	10 (30.3%)	
Type 4 (SS >45°)	12 (40.0%)	9 (27.3%)	
Total	*n* = 30 (100%)	*n* = 33 (100%)	

Results are presented as mean (standard deviation) or count (percent).

^a^ According to the AO Spine thoracolumbar fracture classification, 8 (12.7%) were A3, 4 (6.4%) were A4, 1 (1.6%) were B1, 8 (12.7%) were B2 and 1 (1.6%) were B3 injuries types. SS, sacral slope.

### 3.2 Surgical parameters

ACR was most frequently performed at L3/4 (36.7%) and L4/5 (23.3%) but included all levels in the thoracolumbar region until T11/12 and surgeries entailed multilevel fusions in 50% (3–7 levels) and long constructs in 26.7% (>7 levels; Table 2). ACR procedures were longer (mean 434 vs. 298 min, *p* < 0.001), while estimated blood loss remained comparable (mean 920 ml vs. 610 ml, *p* = 0.099). Intraoperative AEs were comparable with 3.3% of cases in the ACR group and 3.0% in the non-ACR group (*p* = 0.945). No significant disparities were observed between both groups in terms of the type of interbody spacer (*p* = 0.384; Table 2).

**Table 2:** Surgical parameters of patients treated by lateral lumbar interbody fusion (LLIF) using an expandable interbody cage with (w/) or without (w/o) release of the anterior longitudinal ligament (ALL).

Variable	w/ ALL release	w/o ALL release	*p*-value
LLIF segment			0.077
T11-12	1 (3.3%)	3 (7.5%)	
T12-L1	1 (3.3%)	11 (27.5%)	
L1-2	4 (13.3%)	2 (5.0%)	
L2-3	6 (20.0%)	8 (20.0%)	
L3-4	11 (36.7%)	12 (30.0%)	
L4-5	7 (23.3%)	4 (10.0%)	
Number of fused segments			0.443
Mono-/bisegmental	7 (23.3%)	13 (32.5%)	
3–7 segments	15 (50.0%)	14 (35.0%)	
8 or more segments	8 (26.7%)	13 (32.5%)	
Length of surgery, in minutes	434 (138)	298 (166)	<0.001
Estimated blood loss, in milliliters	920 (705)	610 (763)	0.099
Type of interbody cage			0.384
Parallel (0° lordosis)	2 (6.7%)	3 (7.5%)	
Anatomical (6° lordosis)	1 (3.3%)	4 (10.0%)	
Lordotic (5–20° lordosis)	19 (63.3%)	28 (70.0%)	
Hyperlordotic (15–30° lordosis)	8 (26.7%)	5 (12.5%)	
Intraoperative AEs			0.945
No	29 (96.7%)	32 (97.0%)	
Yes, type:	1 (3.3%)	1 (3.0%)	
Vascular injury	–	–	
Nerve injury	–	–	
Cage subsidence	–	–	
Other^a^	1	1	
Total	*n* = 30 patients/*n* = 30 levels	*n* = 33 patients/*n* = 40 levels	

Results are presented as mean (standard deviation, range) or count (percent).

^a^ Complications: asymptomatic cement leakage in 2 patients and cerebrospinal fluid leak in 1 patient.

### 3.3 Radiographic outcomes

As depicted in Table 3, there were no group-differences in terms of sagittal radiological parameters at time of discharge, 3 and 12 months postoperative (*p* > 0.05, except for PT at 12 months), illustrating similarly balanced spines at all time points.

**Table 3:** Sagittal radiological parameters over time of patients treated by lateral lumbar interbody fusion using an expandable interbody cage with (w/) or without (w/o) release of the anterior longitudinal ligament (ALL).

Parameter	Discharge			3 months			12 months		
	w/ ALL release	w/o ALL release	*p*-value	w/ ALL release	w/o ALL release	*p*-value	w ALL release	w/o ALL release	*p*-value
LL, in°	45.2 (8.3)	46.7 (10.2)	0.563	45.8 (13.4)	44.6 (13.6)	0.697	41.9 (15.4)	44.9 (14.7)	0.532
PT, in°	20.4 (7.7)	16.8 (7.4)	0.101	21.6 (8.1)	19.4 (11.7)	0.353	21.9 (8.2)	16.7 (9.7)	0.022
Segmental lordosis, in°	16.4 (13.5)	10.5 (12.3)	0.062	15.0 (13.7)	10.9 (13.7)	0.232	16.9 (14.0)	10.4 (14.4)	0.091
C7 SVA, in cm	5.8 (4.2)	5.4 (4.5)	0.974	5.4 (4.4)	3.7 (3.7)	0.134	7.1 (4.9)	5.9 (5.4)	0.221
Total	*n* = 63 patients/*n* = 70 levels								

Results are presented in degree (°) or centimeters (cm) as mean (standard deviation, range). C7 SVA = C7 sagittal vertical axis; LL = lumbar lordosis; PI, pelvic incidence; PT, pelvic tilt.

**Figure 2:**
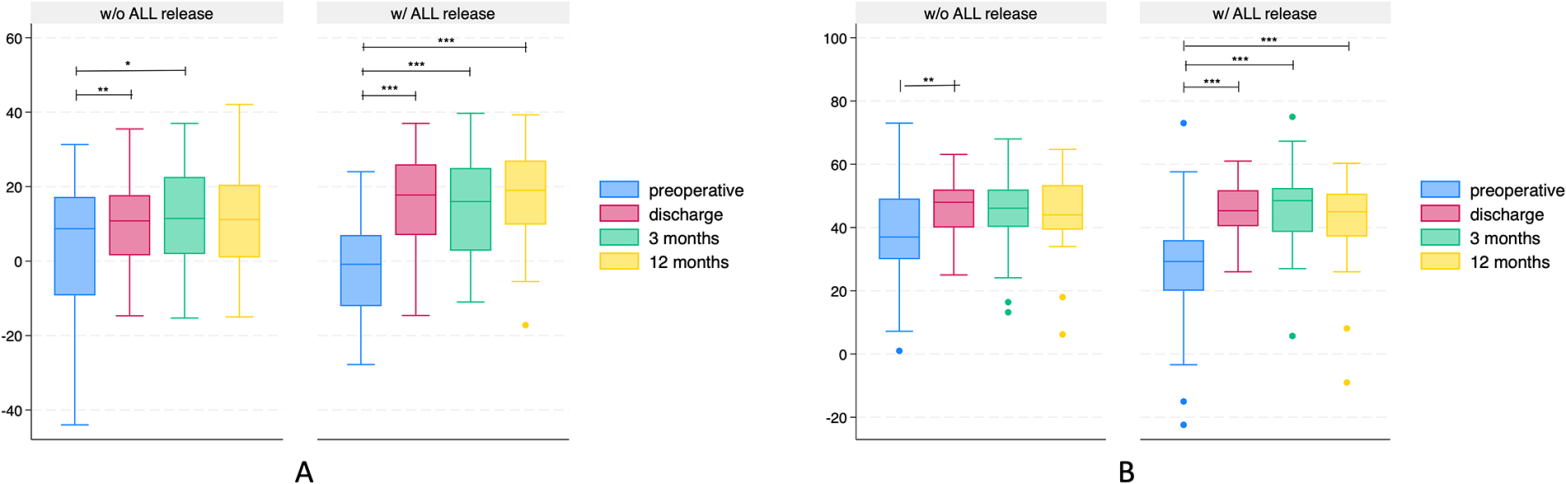
Box plots illustrating lordosis (positive values) or kyphosis (negative values) in degree (°) over time of patients undergoing lateral lumbar interbody fusion with (w/) or without (w/o) release of the anterior longitudinal ligament (ALL). **(A)** Segmental lordosis. **(B)** Total lumbar lordosis. Note that patients in the ALL-release group were more kyphotic preoperative and more lordotic at all time points postoperative, resulting in a more significant segmental correction. *p < 0.05; **p < 0.01; ***p < 0.001.

Given the initially more severe sagittal imbalance of the spine in the ACR group before surgery, there was a highly significant and more substantial correction in sagittal radiological parameters in patients who underwent ALL release, observed consistently across all time points. (Table 4). After ACR, mean SL achieved was 19.2° (95% CI 15.7–22.7°, *p* < 0.001), which remained stable until the 12-month follow-up (18.2°, 95% CI 14.6–21.7°, *p* < 0.001; Figures 2A,B).

**Table 4:** Sagittal radiological parameters over time of patients treated by lateral lumbar interbody fusion using an expandable interbody cage with (w/) or without (w/o) release of the anterior longitudinal ligament (ALL).

Parameter	Discharge		3 months		12 months	
	w/ ALL release	w/o ALL release	w/ ALL release	w/o ALL release	w/ ALL release	w/o ALL release
LL, in°	19.9 (12.4–27.4)***	10.8 (4.6–17.1)**	18.2 (10.7–25.6)***	6.3 (−1.7–14.2)	16.8 (8.1–25.5)***	4.9 (−1.9–11.8)
PT, in°	−6.3 (−2.9 – −9.1)***	−3.7 (1.0 – −8.4)	−5.6 (−3.6 – −7.7)***	−0.2 (7.0 – −7.4)	−5.1 (−3.3 – −7.0)***	−3.5 (2.9 – −9.9)
Segmental lordosis, in°	19.2 (15.7–22.7)***	6.1 (1.8–10.5)**	18.0 (14.0–22.0)***	4.5 (0.5–8.6)*	18.2 (14.6–21.7)***	3.2 (−1.0–7.5)
C7 SVA, in cm	−3.4 (−0.6 – −6.3)*	−3.4 (−0.9 – −6.1)*	−3.1 (−0.4 – −5.8)*	−2.2 (0.1 – −4.6)	−1.9 (−5.5–1.7)	−1.4 (−3.6–0.7)
Total	*n* = 63 patients/*n* = 70 levels					

Results are presented as mean difference from preoperative in degree (°) or centimeters (cm) as mean (95% confidence interval). C7 SVA, C7 sagittal vertical axis; LL, lumbar lordosis; PI, pelvic incidence; PT, pelvic tilt. Level of significance for the difference from pre- to postoperative.

* *p* < 0.05.

** *p* < 0.01.

*** *p* < 0.001.

### 3.4 Complications & outcomes

At discharge, AEs were recorded in 14 (46.7%) patients in the ACR group and 13 (39.4%) patients in the non-ACR group (*p* = 0.560; Table 5). Postoperative AEs were noticed in ten patients (33.3%) of the ACR group and seven patients (21.2%) of the non-ACR group at 3 months (*p* = 0.299). At 12 months, AEs were detected in nine patients (30.0%) of the ACR group and five patients (15.1%) of the non-ACR group (*p* = 0.323). Severity of AEs, according to the TDN grading scale, was comparable between both groups at discharge, 3 and 12 months. Details about the specific type of complication is indicated in Table 5. There was no case of pseudoarthrosis at the LLIF level in any patient or group (*p* > 0.99).

**Table 5:** Complications and clinical outcomes at discharge, 3 and 12 months of patients treated by lateral lumbar interbody fusion using an expandable interbody cage with (w/) or without (w/o) release of the anterior longitudinal ligament (ALL).

Parameter	Discharge			3 months			12 months		
	w/ALL release	w/o ALL release	*p*-value	w/ALL release	w/o ALL release	*p*-value	w/ALL release	w/o ALL release	*p*-value
Functional outcome	n/a	n/a	n/a			0.614			0.622
Excellent				10 (33.3%)	12 (36.4%)		6 (20.0%)	10 (30.3%)	
Good				11 (36.7%)	10 (30.3%)		9 (30.0%)	12 (36.4%)	
Fair				4 (13.3%)	5 (15.1%)		7 (23.3%)	5 (15.1%)	
Poor				4 (13.3%)	2 (6.1%)		3 (10.0%)	1 (3.0%)	
Missing data				1 (3.3%)	4 (12.1%)		5 (16.7%)	5 (15.1%)	
Postoperative AE^a^			0.560			0.299			0.323
No	16 (53.3%)	20 (60.6%)		19 (63.3%)	22 (66.7%)		16 (53.3%)	23 (69.8%)	
Yes	14 (46.7%)^c^	13 (39.4%)^d^		10 (33.3%)^e^	7 (21.2%)^f^		9 (30.0%)^g^	5 (15.1%)^h^	
Missing data	−(0%)	−(0%)		1 (3.3%)	4 (12.1%)		5 (16.7%)	5 (15.1%)	
TDN grading scale			0.094			0.648			0.528
Grade 1	−(0%)	−(0%)		1 (3.3%)	1 (3.0%)		1 (3.3%)	−(0%)	
Grade 2	8 (26.7%)	7 (21.2%)		3 (10.0%)	2 (6.1%)		1 (3.3%)	1 (3.0%)	
Grade 3	2 (6.7%)	6 (18.2%)		6 (20.0%)	4 (12.1%)		6 (20.0%)	3 (9.1%)	
Grade 4	4 (13.3%)	−(0%)		−(0%)	−(0%)		1 (3.3%)	−(0%)	
Grade 5	−(0%)	−(0%)		−(0%)	−(0%)		−(0%)	1 (3.0%)	
Pseudarthrosis^b^	n/a	n/a				>0.99			>0.99
No				30 (100%)	40 (100%)		30 (100%)	40 (100%)	
Yes				−(0%)	−(0%)		−(0%)	−(0%)	
Total	*n* = 30 patients/*n* = 30 levels	*n* = 33 patients/*n* = 40 levels		*n* = 30 patients/*n* = 30 levels	*n* = 33 patients/*n* = 40 levels		*n* = 30 patients/*n* = 30 levels	*n* = 33 patients/*n* = 40 levels	

Results are presented as count (percent).

^a^ Adverse events (AEs) are indicated as occurring between surgery and discharge, discharge, and 3 months, or between the 3- and 12-month follow-up.

^b^ at the LLIF level. TDN = Therapy-Disability-Neurology grading scale of complications.

^c^ Complications: Anemia (*n* = 3), pulmonary embolism (*n* = 3), urinary tract infection (*n* = 2), epidural hematoma requiring posterior revision (*n* = 1), agitation requiring intensive care unit stay (*n* = 1), groin hypoesthesia (*n* = 1), foot drop (*n* = 1), psoas weakness (*n* = 1), Ogilvie syndrome (*n* = 1).

^d^ Complications: Anemia (*n* = 4), (transient) foot drop (*n* = 2), acute coronary syndrome (*n* = 1), clostridium difficile infection (*n* = 1), delirium (*n* = 1), pneumonia (*n* = 1), pneumothorax (*n* = 1), sepsis (*n* = 1), psoas weakness (*n* = 1).

^e^ Complications: Surgical site infection requiring revision (*n* = 2), proximal junctional kyphosis (*n* = 2), groin hypoesthesia (*n* = 1), pseudarthrosis at L5/S1 (*n* = 1), foot drop (*n* = 1), osteoporotic compression fracture of distant vertebral body (*n* = 1), new hip pain (*n* = 1), psoas weakness (*n* = 1).

^f^ Complications: New foot drop (*n* = 2), ilisacral joint pain (*n* = 2), posterior wound infection (*n* = 1) or dehiscence (*n* = 1), psoas-weakness (*n* = 1).

^g^ Complications: Proximal junctional kyphosis or failure (*n* = 4), low-grade infection (*n* = 2), asymptomatic screw loosening (*n* = 1), pseudarthrosis requiring revision (*n* = 1), ilisacral joint pain (*n* = 1).

^h^ Complications: Proximal junctional kyphosis (*n* = 1), lethal myocardial infarction (*n* = 1), LLIF access-related psuedohernia (*n* = 1), adjacent segment disease (*n* = 1), bursitis trochanterica (*n* = 1).

There was no difference in outcome at 3 and 12 months of follow-up (Table 5). Most patients, comprising *n* = 25 (83.3%) in the ACR group and *n* = 27 (81.8%) in the non-ACR group, demonstrated excellent to fair functional outcomes at the 3-month follow-up. At the 12-month follow-up, *n* = 22 (83.3%) patients in the ACR group and *n* = 27 (81.8%) patients in the non-ACR group sustained outcomes falling within the excellent to fair range.

### 3.5 Logistic regression analysis

In univariable analysis, patients undergoing ACR with ALL release were as likely as patients without ALL release to experience any complication until 12 months postoperative (OR 1.92, 95% CI 0.61–6.09, *p* = 0.263), any surgical complication until 12 months (OR 2.00, 95% CI 0.67–5.98, *p* = 0.215) or favorable outcome (OR 0.41, 95% CI 0.12–1.36, *p* = 0.146; Table 6). Effect sizes were slightly adjusted in multivariable models, remaining without significant effect of ALL release on any of these three outcomes (Table 6).

**Table 6:** Uni- and multivariable logistic regression analysis, estimating the relationship between anterior column realignment (ACR) by deliberate release of the anterior longitudinal ligament (ALL) and a) occurrence of any complication until 12 months postoperative, b) occurrence of any surgical complication until 12 months postoperative, or c) favorable (excellent/good) clinical outcome at 12 months. Results are presented as odds ratios (OR) with 95% confidence interval (CI), adjusted for baseline differences in the American Society of Anesthesiology (ASA) risk scale and indication for surgery.

		Univariable model			Multivariable model		
		OR	95% CI	*p*-value	OR	95% CI	*p*-value
Any complication until 12 months	ALL release/ACR	1.92	0.61–6.09	0.263	1.83	0.50–6.68	0.360
	ASA grade^a^	1.29	0.63–2.63	0.480	1.18	0.56–2.50	0.650
	Primary indication^b^	1.12	0.74–1.69	0.579	0.99	0.62–1.59	0.973
Any surgical complication until 12 months	ALL release/ACR	2.00	0.67–5.98	0.215	1.97	0.57–6.76	0.280
	ASA grade^a^	1.05	0.53–2.08	0.890	0.93	0.45–1.93	0.858
	Primary indication^b^	1.13	0.76–1.68	0.520	1.03	0.66–1.61	0.892
Favorable outcome at 12 months	ALL release/ACR	0.41	0.12–1.36	0.146	0.51	0.13–1.95	0.324
	ASA grade^a^	0.39	0.16–0.95	0.038	0.40	0.15–1.04	0.061
	Primary indication^b^	0.79	0.52–1.21	0.297	0.95	0.58–1.55	0.851

^a^ Categorical variable – per each 1-step increase in ASA grade.

^b^ Categorical variable, including trauma, deformity, revision surgery, degenerative disc disease and other (infections, etc.).

## 4 Discussion

Historically, the restoration of lordosis in ASD involved the utilization of extensive multicolumn osteotomies. Schwab et al. (28) have introduced a comprehensive classification system for posterior osteotomies, categorizing them according to increasing complexity, destabilization, and gains in SL. Among these classifications, three-column osteotomies, including PSO and VCR, are noted for yielding the highest augmentation of SL, frequently surpassing 20–25° per level. However, these procedures are associated with significant morbidity (7). A recent historical review of 573 patients undergoing a three-column osteotomy revealed that the incidence of major complications was 39% and blood loss exceeding 4l occurred in 16.7% of patients (29).

**Figure 3:**
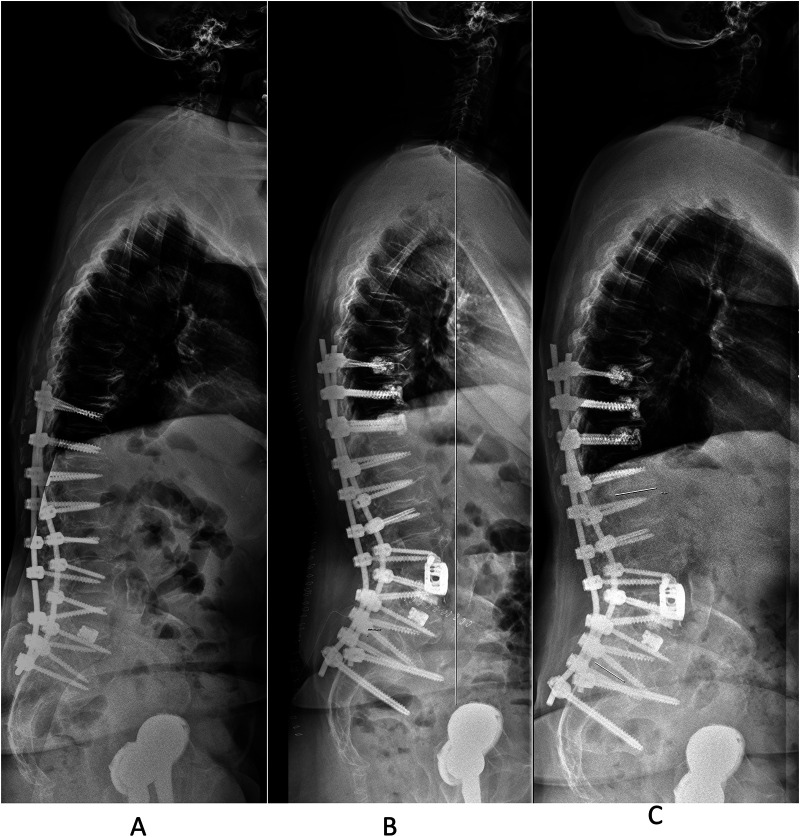
A 69-year-old female presented with pseudoarthrosis at the L3/L4 and T12/L1 levels, along with lumbar “flat back”, coronal deformity and rod fracture L3/4. She has a history of spinal surgery for degenerative scoliosis with attempted fusion between T10-S1 with TLIF at L5/S1, performed in an outside hospital in the region. **(A)** Preoperative standing lateral x-ray studies demonstrate a pelvic incidence of 63.1°, corresponding to a Roussouly type 4 spinal geometry. Her total lumbar lordosis (LL) was 24.8°, pelvic tilt (PT) 46.3°, sacral slope (SS) 28.9° and segmental lordosis (SL) at L3/4 6°. We performed a revision posterior instrumented fusion from T9-S2Ai using cement-augmented screws at T9-11 and L4. Anterior column realignment via a LLIF-trajectory was performed at the L3/4 level, employing an expandable 15-30° ELSA® spacer and bilateral SPOs. **(B)** Lateral standing x-ray studies before discharge with evidence of 34.5° SL at L3/4 (increase in SL of 28.5°), LL of 46.2°, SS of 30.2°, PT of 37.1° **(C)** Lateral standing x-ray studies at 1-year follow-up, with minimal loss of SL to 33.4° and LL to 43° (SS 33° and PT 42.1°) in a patient reporting an excellent clinical outcome.

In view of this morbidity, the technique of ACR evolved in the past years with newly designed instruments and retractors making it safer and more re-producible. Lateral approaches to the anterior and middle spinal column typically rely on the integrity of the ALL to manage graft tension and hinder the anterior displacement of interbody cages. Yet, in the context of correcting sagittal deformities, the ALL serves as a crucial barrier to anterior column elongation and deformity correction. Hence, ACR involves controlled release of the ALL, alongside inserting a (hyperlordotic) cage to address localized kyphotic spinal deformities. The introduction of expandable LLIF cages that can be fixed to the adjacent vertebrae offer attractive solutions, especially suited for ACR (Figures 1, 3). In this article we review our institutional series of the past years while applying these types of implants.

### 4.1 Radiological parameters

Our results demonstrate that ACR with ALL release, conducted in MIS fashion, allows for powerful correction of segmental and global sagittal deformities. Compared to LLIF without ALL release (10.8°), ACR resulted in twice as much segmental correction (19.2°) immediately after surgery, which was grossly maintained during the 12-month follow-up (18.2°; Table 4). This falls within the range of correction obtained with traditional posterior three-column osteotomies that typically amount to 15–35° (30–32). Our findings are consistent with a recent review of the literature, where an increase in SL between 12° and 39° was reported after ACR (33). Our results likewise compare well with a recent study by Jeon et al. (34), who applied an anterior-to-psoas ACR technique with partial ALL release in combination with PCOs to gain overall increase in SL of 15.8° ± 6.7° at follow-up. Manwaring et al. (35) reported on their experience with two-staged surgery, where patients underwent LLIF with (*n* = 9) or without ACR (*n* = 27), followed by a delayed second stage of percutaneous posterior spinal instrumentation. The authors achieved an increase in total LL by 16.5° and in SL by 12° per ACR level.

We further noticed an excellent fusion rate, with no case of pseudarthrosis identified at the LLIF level. In accordance with the recently published work by Herase et al. (36), no cases of pseudarthrosis were observed in their ACR group, either. In other comparable articles, fusion rates following ACR ranged between 92.9% (36), 94.6% (34) and 97.9% (37), as determined on postoperative radiographs or CT scans. Compared to this, pseudarthrosis is a more frequent complication and constitutes one of the most relevant indications for revision surgery following PSO, with rates ranging between 12% and 29% (38–41). According to the aforementioned information, the postoperative fusion rate after PSO is approximately set at 70%–80% (41, 42). This rate may be improved by enhancing anterior axial load transfer through the use of interbody cages, spacers, or grafts implanted immediately above and/or below the PSO level (42).

Also, the results in Table 4 demonstrate that SL at the LLIF level was maintained during follow-up with/without ALL release (1°/2.9° loss of SL), whereas some of the total LL was lost over time (3.1°/5.9°; Table 4), mostly not at the level treated by LLIF with the expandable spacer, however (compare Figure 3). This gradual loss of initial correction is well-known in the literature after various treatment modalities, including posterior instrumentation with or without osteotomies and interbody fusion (43). ACR in addition with PCOs appear highly effective in optimizing spinopelvic parameters to the desired degree. As such, ACR is a valid option for ASD in the middle and upper lumbar spine, which may allow to compensate for the lack of lordosis at the lower lumbar levels to some degree (14). Establishing a sustainable & physiologic spinopelvic harmony in ASD patients may help to minimize the risk for adjacent segment disease or proximal junctional failure.

### 4.2 Complications

The LLIF approach for ACR is relatively novel and the learning curve may initially be high, in particular for surgeons not performing LLIF on a regular basis. As surgeons with fairly frequent use of LLIF in daily practice for degenerative disc disease, deformity, trauma, tumors and infections, we noticed intraoperative AEs in about 3% of patients in both study groups. Life-threatening AEs resulting from vessel or bowel injury have been reported (44–46), but such complications were not encountered in our series. In fact, the two intraoperative AEs we encountered were asymptomatic cement leakage (*n* = 2) and cerebrospinal fluid leak (*n* = 1), associated with the posterior part of the surgery.

AEs at time of discharge predominantly comprised medical complications such as anemia (*n* = 7), pulmonary embolism (*n* = 3), and extraspinal infections (*n* = 5). In the ACR group, there was a single case of spinal epidural hematoma, necessitating posterior revision surgery. In the ACR group, the average surgical time was 434 min, aligning well with existing literature documenting surgical times in the range from 400 to 600 min (14). The surgical times for PSO procedures, as reported in the literature, equally fall between 300 and 600 min (47–49). As all LLIF procedures in this series were performed in lateral decubitus position, the re-positioning of the patient to prone position for the posterior approach afterwards is responsible for about 45–60 min of the surgical time. The estimated blood loss (EBL) in this series was 920 ml on average, which appears to be less compared to traditional three-column osteotomy such as PSO (50, 51), where EBL in the range of 1,428 ml (49) to 5,800 ml (48, 52) was reported. In a recently published comparative study of the International Spine Study Group, patients undergoing ACR experienced notably lower EBL compared to patients undergoing PSO (1.6l vs. 3.6l, respectively) with similar correction of spino-pelvic parameters (50).

The rate of AEs at 3 months after ACR (33%) was comparable to the non-ACR group (*p* = 0.299). According to the TDN grading scale, no lethal or life-threatening AEs were encountered at this time, but AEs of moderate severity (grade 3) occurred in one out of five treated ACR patients (Table 5). In fact, the vast majority of AEs noted in our patients at 3 months were medical complications, including anemia, delirium, pulmonary embolism or urinary tract infections. As ACR via a LLIF trajectory involves splitting or retraction of the psoas muscle, there is a potential for injury to the femoral nerve and lumbar plexus (53). Many studies lack sufficient details regarding the anatomic source, severity of injury, and specific ACR technique used, including whether neuromonitoring was employed or not. Consequently, reported rates of sensory (0.7%–30%) and motor deficits following LLIF (3.4%–23.7%) vary broadly (53–55). In our series, three patients (4.8%) reported psoas- or lumbar plexus-related morbidity (groin hypoesthesia or hip weakness), all of which recovered over time. One additional patient complained about a pseudohernia at the approach-side, which was treated surgically by mesh-repair.

Between 3 and 12 months, further AEs were noticed in 30% of the ACR group, which was again comparable to the non-ACR group (*p* = 0.323). One patient suffered from myocardial infarction and died about 4 months postoperative, unrelated to the surgery. Other reasons for delayed AEs included iliosacral or hip joint pain, low-grade infections, pseudarthrosis (at other spinal segments), adjacent segment disease or proximal junctional kyphosis/failure.

Altogether, the rates of AEs in our series fall within the range of complication rates reported in similar cohort studies on ACR, which varied from 0% to 61.3% (14, 44, 56, 57), encompassing transient and persistent motor and sensory deficits, radiculopathy, proximal junctional kyphosis, vascular injury, dural tear or cerebrospinal fluid leak, ileus, retroperitoneal hematoma, fracture, and wound complications. The AE rates after ACR are also comparable to those after traditional posterior three-column osteotomies, ranging from 24.2% to 50.8% (58, 59). Due to the lack of comprehensive severity grading of AEs in previous studies, the impact of AEs on a patients’ well-being cannot be compared between series or surgical techniques (60). Characterizing the true complication rates following ACR and their clinical significance remains challenging.

### 4.3 Outcomes

Our results demonstrate fair, good or even excellent outcome in the majority of patients at the 12-month follow-up, with no statistical difference between the ACR (83.3%) and the non-ACR group (81.8%; *p* > 0.05), despite the larger degree of correction. The available literature on clinical outcomes after ACR is still limited (14, 35, 44, 50, 57, 61–64). In one of the larger series so far (*n* = 39), Hosseini et al. (57) described an improvement in visual analog scale (VAS) for back pain from 6.8 pre- to 3.0 postoperatively, further improving to 2.1 at 12 months. Correspondingly, the Oswestry disability index (ODI) decreased from 54% to 38%, with further improvement to 26% at 12 months. Considering this report's challenging population of patients with severe deformities, failed previous fusion surgeries and significant medical comorbidities, the overall rate of fair to excellent outcome appears adequate.

### 4.4 Strengths and limitations

This series of consecutively treated patients at a tertiary regional reference center provides new data on a relatively novel technique, for which the current literature is limited. We provide comprehensive radiological, complication and outcome data until 12 months postoperative in a reasonably large series of ACR patients treated via LLIF approach and by use of an expandable interbody spacer. In contrast to single-surgeon series, our results obtained by various surgeons from our team allow for a better generalization of results. Moreover, we included a control group of patients undergoing LLIF with the same type of spacer but without ALL release, which helps to isolate the effect of the latter.

Limitations include the study's retrospective nature, missing data for some of the variables (indicated in the tables) and a mean follow-up period of only about 12 months. These factors may also limit the validation of cage subsidence and cage integrity over time, as well as the impact of ALL release on these complications. A further limitation of this study is the absence of standardized CT scans for the assessment of the fusion status, as those are not routinely performed as part of the follow-up regimen in patients faring well. However, in case of unusual pain during follow-up, CT or SPECT scans were ordered to rule out pseudarthrosis. To categorize the clinical outcome, simple MacNab criteria were applied because the standardized use of patient-reported outcome measures (PROMs) was only introduced in our center in 2022 and was missing for a significant proportion of patients. This is a shortcoming, as prior studies showed a considerable rate of back pain after ACR using a lateral approach (65), and a more objective way of determining change in pain & disability scores pre- to postoperative would have been helpful. Careful consideration is warranted regarding the inherent limitations associated with the physician-rated and subjective scoring system for the functional outcome. Despite thorough documentation intraoperatively (written report & intraoperative x-rays taken), we cannot exclude accidental ALL release in the control group with 100% certainty. Should there be any case with unintended ALL release in the control group, the true difference in SL between ACR and non-ACR segments could even be higher than reported here.

## 5 Conclusions

ACR using an expandible LLIF interbody implant was safe, promoted solid fusion and restored significantly more segmental lordosis compared to LLIF without ALL release, which was maintained during follow-up.

## Data Availability

The raw data supporting the conclusions of this article will be made available upon reasonable request.
